# Over-indebtedness and its association with the prevalence of back pain

**DOI:** 10.1186/1471-2458-9-451

**Published:** 2009-12-06

**Authors:** Elke B Ochsmann, Heiko Rueger, Stephan Letzel, Hans Drexler, Eva Muenster

**Affiliations:** 1 Institute of Occupational and Social Medicine, Medical Faculty, RWTH Aachen University Pauwelsstrasse 30, D-52074 Aachen, Germany; 2 Institute of Occupational, Social, and Environmental Medicine, University of Erlangen-Nuremberg, Schillerstrasse 25, D-91054 Erlangen, Germany; 3 Institute of Occupational, Social, and Environmental Medicine, University of Mainz, Obere Zahlbacher Strasse 67, D-55131 Mainz, Germany

## Abstract

**Background:**

Over-indebtedness is an increasing phenomenon worldwide. Massive financial strain, as found in over-indebted persons, might influence the occurrence of back pain. In this explorative study we examined the prevalence of back pain in over-indebted persons in Germany for the first time ever and compared it to the prevalence of back pain in the German general population.

**Methods:**

A cross sectional study comprising 949 participants (52.6% women) was conducted to collect data on the point prevalence of back pain in an over-indebted collective. A representative sample of the German general population (N = 8318, 53.4% women) was used as non-indebted reference group.

**Results:**

The point prevalence of back pain was 80% in the over-indebted collective, compared to 20% in the general population. The influence of socioeconomic factors on the prevalence of back pain differed partially between the general population and the over-indebted collective. Being over-indebted was identified as an independent effect modifier and was associated with an eleven times increased probability to suffer from back pain (aOR: 10.92, 95%CI: 8.96 - 13.46).

**Conclusion:**

Until now, only little is known about the effects of intense financial strain like over-indebtedness on health. Our study suggests that over-indebted persons represent a risk group for back pain and that it might be sensible to take financial strain into account when taking a medical history on back pain. Over-indebtedness and private bankruptcy is of increasing importance in industrialized countries, therefore more research on the subject seems to be necessary.

## Background

It stands to reason that the worldwide financial crisis increases a phenomenon known as "over-indebtedness". With the provision of new products in financial services, access to loans is easier than ever, consumer over-indebtedness is increasing, and this alerts the European public to this new upcoming risk [1]. There is currently no standard definition of over-indebtedness which is accepted throughout the European Union. In Germany, a private household is said to be over-indebted, if "its income over an extended period is not sufficient for servicing debt on time (after deducting costs of living expenses) despite a reduction of the standard of living" [1]. It is estimated that currently 3.13 million private households in Germany alone are affected by over-indebtedness [2]. But over-indebtedness does not seem to be exclusively a European problem. For example, in 2004 12.1% of U.S. citizens were at least in danger of being over-indebted (considering the official poverty rate) [3], a number that - regarding the current financial markets - probably has to be adjusted upwards. Over-indebted private households are in a fragile economic situation and are in danger of social exclusion and increased vulnerability [4]. Until now only little is known about extensive financial strain like over-indebtedness and its consequences to health. In another evaluation, we were able to describe an association between over-indebtedness and obesity for the first time [5]. Apart from obesity, back pain is also a health condition which was reported to be associated with psychosocial factors. Perceived financial strain could represent a moderator variable for the socio-economic position in adult life which again has been reported to be associated with the prevalence of low back pain [6-9]. Therefore, this study deals with the possible association between over-indebtedness and the prevalence of back pain in a German over-indebted cohort.

### Aim

As the prevalence of back pain was reported to be associated with the social status of a person, we hypothesized that over-indebtedness, i. e. being under strong financial strain, might be associated with an increased prevalence of back pain and might pose an effect modifier of back pain. In order to elucidate this hypothesis we compared a cohort of over-indebted persons to a representative sample of the German general population.

## Methods

### Over-indebted individuals (OI-survey)

A cross-sectional study on over-indebted individuals (OI-survey) of the University of Mainz considered over-indebted persons in Germany (older than 16 years) who sought out free-of-charge debt counselling agencies. The survey was carried out by these debt counselling agencies of Rhineland-Palatinate and Mecklenburg-Western Pomerania. One person per over-indebted household, usually the one seeking advice, was asked to fill in a self administered questionnaire on over-indebtedness, psychosocial and socioeconomic factors and health. Amongst other things, the questionnaire covered the following issues: current medical problems (in this evaluation we considered "mental illnesses at the moment (e. g. depression)", and "back pain at the moment"), social support (using e. g. a standardized German questionnaire for social support [10-12]) and questions on over-indebtedness (e. g. duration, amount of debt, legal situation). The questionnaire was evaluated and improved after a feasibility study in 2006. Details on the survey have recently been published [12,13]. Altogether 2,711 copies of the final questionnaire as well as prepaid envelopes were handed out to over-indebted consulters during one of their counselling sessions and the consulters were asked to send back the filled-in questionnaire anonymously to the study centre at the University of Mainz. Without any reminder action, altogether 949 persons (response rate: 35.0%) participated in the OI-survey.

### German general population (German National Telephone Health Interview Survey 2003 - GNT-HIS)

Data of a representative sample of the German general population was obtained by a telephone interview conducted by the Robert-Koch-Institute in 2003 (GNT-HIS). Again, details on the survey have already been published [14,15]. In the GNT-HIS, 15,918 persons were contacted via telephone, 8,318 individuals participated (response rate: 52.3%). The interview questionnaire covered various aspects of diseases (e. g. "Do you suffer from depression"), including risk factors for these diseases, quality of life, health care utilisation and socioeconomic status.

### Low back pain

The question "To what extent do you complain about back pain at the moment? (five possible answers)" was used to identify the point prevalence of LBP in the over-indebted collective (OI-survey). To compare the OI-data with the GNT-HIS-data, the five possible answers were dichotomized. "I complain to some degree", "I complain considerably" and "I complain strongly" were encoded as "back pain", while " I do not complain" and " I barely complain" were encoded as "no back pain". In the GNT-HIS, the point prevalence of LBP was calculated according to the question in the GNT-HIS 2003-questionnaire "Did you have back pain yesterday" with the possible answers "yes" and "no". Chronologically all persons participating in the GNT-HIS were firstly asked whether they experienced LBP during the last twelve months: "Did you experience back pain during the last 12 months?". Secondly, only those persons who had stated to have had LBP during the last 12 months were questioned further whether they experienced LBP yesterday.

### Other variables

In both data sets, age was stratified into four groups (<= 30 years, 31-40 years, 41-50 years, and >= 51 years). Data on socioeconomic variables was collected by ticking the corresponding answers, which were analogue in the two data sets. Apart from that, physical activity and smoking behaviour were surveyed by similar pre-formulated answers in the two data sets, too. Body-Mass-Index was calculated by self-reported height and weight and stratified into "underweight and normal weight", "overweight" and "obesity" according to the WHO-definition [16]. Mental illnesses in the OI-survey were evaluated by the questioning "Do you suffer from mental illnesses (e.g. panic attacks, depression)", while in the GNT-HIS the question was solely focused on depression: "Do you suffer from depression?".

### Combined data set

In order to evaluate the possible association between being over-indebted and the prevalence of back pain, both data sets (OI-survey and GNT-HIS) were combined. All participants of the GNT-HIS were categorised as "not over-indebted", while all OI-participants were categorised as "over-indebted". Although a bias towards the null cannot be ruled out, this procedure was chosen due to lack of information on debts in the GNT-HIS.

### Statistical methods

In both data sets, the prevalence of low back pain associated with over-indebtedness was calculated using SPSS 15.0 (Microsoft). As potential confounders from the literature sex, age, school and professional education, status of employment, mental illnesses (especially depression), Body-Mass-Index, physical activity and smoking habits were considered. Bivariate group differences were tested by calculating the unadjusted odds ratio. Continuous variables were categorized, to find non-linear effects. Multivariate analyses (multivariate binary logistic regression model (inclusion)) were conducted. P < 0.05 was considered to be statistically significant. Adjusted odds ratio (aOR) and associated 95% confidence intervals (95%CI) were calculated.

### Ethical approval

The ethical committee of the medical association of the German Bundesland Rhineland-Palatine and the data protection officer of Rhineland-Palatinate approved the OI-survey in the German states Rhineland-Palatinate and Mecklenburg-Western Pomerania. Approval of an ethical committee was not necessary for the GNT-HIS data, as these were an existing public access dataset on which only secondary analyses were conducted.

## Results

### Over-indebted participants of the OI-survey

All in all 949 persons (response rate: 35%), 446 male (47.0%) and 499 female (52.6%), aged between 18 and 79 years (41.27 ± 11.31 years, median: 41 years) participated in the OI-survey. 767 of them (80.8%) reported to have had back pain at the moment of being questioned.

### German general population of the GNT-HIS

Of 15.918 persons who were contacted via telephone, 8318 persons participated in the GNT-HIS (recourse: 52.3%), 3872 male (46.5%) and 4446 female (53.4%), aged between 18 and 96 years (46.67 ± 15.64, median 45 years), 22.2% (n = 1849) of whom reported to have had LBP yesterday.

### Back pain prevalence of over-indebted persons in comparison to the general population - potential factors of influence

Unadjusted odds ratios for potentially influencing factors known from the literature are depicted in tables 1, 2 and 3. In the general population, a higher risk for reporting back pain was observed for female participants, age above 40 years, being married and living together, lower educational and professional status, retirement, unemployment or being a homemaker, lack of physical activity, overweight or obesity, and depression. In the over-indebted population, a higher risk for reporting back pain was associated with the following factors: age above 30 years, being married and living together, lack of physical activity, and reporting mental illnesses like depression.

**Table 1:** Association between biometric data and back pain in the over-indebted OI-cohort and the general population of the GNT-HIS

	OI-survey (n = 949)						GNT-HIS (n = 8318)					
	**Total**		**Back pain**		**Unadjusted odds ratios**		**Total**		**Back pain**		**Unadjusted odds ratios**	
	**n = 949**	**%**	**n = 767**	**%**	**OR**	**95%CI**	**n = 8318**	**%**	**N = 1849**	**%**	**OR**	**95%CI**

**Sex**												

*male*	446	47.0	360	80.7			3872	46.5	663	17.1		
*female*	499	52.6	406	81.4	1.04	0.75-1.44	4446	53.5	1186	26.7	**1.76**	**1.58-1.96**

**Age**												

*<= 30 years*	199	21.0	139	69.8			1324	15.9	223	16.8		
*31-40 years*	244	25.7	204	83.6	**2.20**	**1.40-3.47**	1829	22.0	344	18.8	1.14	0.95-1.38
*41-50 years*	301	31.7	257	85.4	**2.52**	**1.62-3.92**	2027	24.4	443	21.9	**1.38**	**1.16-1.65**
*>= 51 years*	201	21.2	166	82.6	**2.05**	**1.28-3.29**	3138	37.7	839	26.7	**1.80**	**1.53-2.12**

**Bold print: **statistically significant results; level of significance p < 0.05

**Table 2:** Association between socioeconomic variables and back pain in the over-indebted OI-cohort and the general population of the GNT-HIS

	OI-survey (n = 949)						GNT-HIS (n = 8318)					
	Total		Back pain		Unadjusted odds ratios		Total		Back pain		Unadjusted odds ratios	
	n = 949	%	n = 767	%	OR	95%CI	n = 8318	%	N = 1849	%	OR	95%CI
**Nationality**												

*German*	904	95.3	735	81.3			8018	96.4	1784	22.2		
*other*	33	3.5	25	75.8	0.72	0.32-1.62	300	3.6	65	21.7	0.97	0.73-1.28

**Family status**												

*married and living together*	281	29.6	236	84.0			4639	55.8	1070	23.1		
*married and living alone*	71	7.5	59	83.1	0.94	0.47-1.88	203	2.4	51	25.1	1.12	0.81-1.55
*single*	297	31.3	222	74.7	**0.56**	**0.37-0.85**	2126	25.6	357	16.8	**0.67**	**0.59-0.77**
*divorced*	256	27.0	212	82.8	0.92	0.58-1.45	692	8.3	187	27.0	**1.24**	**1.03-1.48**
*widowed*	37	3.9	33	89.2	1.57	0.53-4.66	640	7.7	179	28.0	**1.30**	**1.08-1.56**

**School education**												

*Hauptschule e. g. expanded primary school*	461	48.6	378	82.0			2412	29.0	658	27.3		
*no graduation*	95	10.0	72	75.8	0.69	0.41-1.16	64	0.8	20	31.3	1.21	0.71-2.07
*secondary school*	256	27.0	212	82.8	1.06	0.71-1.58	2167	26.1	488	22.5	**0.78**	**0.68-0.89**
*technical college orgrammar school*	84	8.9	67	79.8	0.87	0.48-1.55	3383	40.7	625	18.5	**0.60**	**0.53-0.68**

**Professional education**												

*apprentice-ship*	462	48.7	380	82.3			2927	35.2	692	23.6		
*no professional education*	237	25.0	189	79.7	0.85	0.57-1.26	745	9.0	219	29.4	**1.35**	**1.12-1.61**
*vocational school*	82	8.6	65	79.3	0.83	0.46-1.48	1133	13.6	288	25.4	1.10	0.94-1.29
*university*	89	9.4	76	85.4	1.26	0.67-2.38	2735	32.9	518	18.9	**0.76**	**0.66-0.86**

**Status of employment**												

*full-time*	210	22.1	173	82.4			3809	45.8	698	18.3		
*apprentice-ship*	18	1.9	15	83.3	1.067	0.30-3.88	245	2.9	30	12.2	**0.62**	**0.42-0.92**
*(early) retirement*	101	10.6	86	85.1	1.23	0.64-2.36	1713	20.6	474	27.7	**1.71**	**1.49-1.95**
*unemployed*	290	30.6	232	80.0	0.86	0.54-1.35	323	3.9	102	31.6	**2.06**	**1.60-2.64**
*homemaker*	148	15.6	114	77.0	0.72	0.43-1.21	685	8.2	188	27.4	**1.69**	**1.40-2.03**
*part-time*	107	11.3	88	82.2	0.99	0.54-1.82	1535	18.5	357	23.3	**1.35**	**1.17-1.56**

**Bold **print: statistically significant results; level of significance p < 0.05

**Table 3:** Association between lifestyle and medical factors and back pain in the over-indebted OI-cohort and the general population of the GNT-HIS

	OI-survey (n = 949)						GNT-HIS (n = 8318)					
	Total		Back pain		Unadjusted odds ratios		Total		Back pain		Unadjusted odds ratios	
	n = 949	%	n = 767	%	OR	95%CI	n = 8318	%	N = 1849	%	OR	95%CI
**Smoking behaviour**												

*daily*	537	56.6	431	80.3			2216	26.6	491	22.2		
*sometimes*	61	6.4	51	83.6	1.25	0.62-2.55	602	7.2	135	22.4	1.02	0.82-1.26
*ex-smoker*	168	17.7	141	83.9	1.28	0.81-2.04	2238	26.9	509	22.7	1.03	0.90-1.19
*non-smoker*	174	18.3	138	79.3	0.94	0.62-1.44	3260	39.2	713	21.9	0.98	0.87-1.12

**Sports/work-out**												

*no sports*	520	54.8	423	81.3			3027	36.4	760	25.1		
*<1 hour/week*	266	28.0	229	86.1	1.42	0.94-2.14	828	10.0	206	24.9	0.99	0.83-1.18
*1-2 hours/week*	78	8.2	65	83.3	1.15	0.61-2.16	1292	15.5	277	21.4	**0.81**	**0.70-0.95**
*>2 hrs/week*	67	7.1	40	59.7	**0.34**	**0.20-0.58**	3134	37.7	597	19.0	**0.70**	**0.62-0.79**

**BMI**												

*underweight and normal weight*	401	42.3	324	80.8			4378	52.6	887	20.3		
*over-weight*	306	32.2	234	76.7	0.79	0.55-1.13	2830	34.0	627	22.3	**1.14**	**1.02-1.28**
*obesity*	238	25.1	206	86.6	1.53	0.98-2.39	938	11.3	286	30.5	**1.73**	**1.48-2.02**

**4Mental illness (e. g. depression)**												

*no*	580	61.1	449	77.4			7529	90.5	1528	20.3		
*yes*	369	38.9	318	86.2	**1.82**	**1.28-2.59**	783	9.4	319	40.7	**2.70**	**2.32-3.15**

**Bold print: **statistically significant results; level of significance p < 0.05

### Influence of the factor "over-indebtedness" on the point prevalence of back pain

After adjustment for all of the above mentioned variables, "being over-indebted" turned out to have an independent effect on the prevalence of back pain (aOR: 10.92, 95%CI: 8.96-13.46) (table 4).

**Table 4:** multivariate analysis of the combined data set (OI-survey and GNT-HIS) with over-indebtedness as independent variable (statistically significant associations only)

	Combined data set (OI-survey and GNT-HIS) (n = 9267)			
	
			adjusted odds ratios	
			aOR	95%CI
**Biometric variables**	*sex*	male	-	-
		female	1.60	1.42-1.80
	
	*Age*	<= 30 years	-	-
		>= 51 years	1.38	1.11-1.72

**Socioeconomic factors**	*school education*	"Hauptschule", expanded primary school	-	-
		technical college or grammar school	0.77	0.67-0.90
	
	*family status*	married and living together	-	-
		single	0.83	0.71-0.97
	
	*status of employment*	full-time	-	-
		unemployed	1.29	1.03-1.62

**Lifestyle/medical factors**	*mental illness (e. g. depression)*	no	-	-
		yes	2.36	2.04-2.74
	
	*physical activity*	no sports	-	-
		>2 hrs/week	0.81	0.71-0.91
	
	*BMI*	underweight and normal weight	-	-
		obesity	1.51	1.29-1.77

**Financial strain**	*over-indebtedness*	no	-	-
				
		yes	10.92	8.96-13.46

* multivariate model (inclusion) adjusted for all variables depicted in tables 1, 2 and 3; table 4 depicts statistically significant associations only; level of significance p < 0.05

## Discussion

In this explorative analysis, over-indebted individuals were more likely to report back pain than individuals of the general population. This association was not fully explained by traditional socioeconomic variables. Therefore, the variable over-indebtedness seems to be an independent predictor of back pain.

In this analysis, the back pain point prevalence of ~20% found in the German general population goes along with the majority of reports which point out varying estimates of back pain point prevalence in the general population ranging from 14 to 28% [17-21]. By contrast the point prevalence of back pain in the over-indebted OI-survey turned out to be approximately 80%. In 2006 Burton et al. stated in the European guidelines for prevention in low back pain that studies are needed to determine how and by whom interventions are best delivered to specific target groups [22]. With an increasing number of over-indebted households worldwide, and with our results in mind, over-indebted persons might pose an emerging risk group for back pain and apart from this first analysis more specific research in this area might be helpful for addressing the back pain problem in this risk group in the near future.

A couple of methodological issues have to be considered with regard to this evaluation. Firstly, the OI-survey was a written questionnaire study, while the GNT-HIS was conducted by a telephone interview. Schwarz et al. [23] reported differences between the results of written and interview questionnaires. The mode of data collection may influence respondents' judgemental processes via its impact on the retrieval of relevant information from memory, its impact on respondents' elaboration of the response alternatives presented to them, and its impact on the judgemental strategies used. Nevertheless, because of the careful interview design, we believe that the differences because of the different administration mode might be only limited. The possible truncation of the memory search process in the GNT-HIS interview, e. g., as compared to the self-administered questionnaire might not be of the utmost importance as the questions referred to back pain yesterday (as compared to "back pain at the moment") which lies in the near past. But secondly, this difference in the definition of back pain point prevalence in this explorative comparison poses another risk for bias. Questioning for "back pain at the moment", which stands for a broader time span, might lead to a higher prevalence than questioning for "back pain yesterday". Nevertheless, when we compared "back pain at the moment" in the over-indebted individuals with "back pain during the last 12 months" in the general population (1-year prevalence), we still calculated elevated adjusted odds ratios of about three. Therefore, we assume that the differences in the methodological approach are not solely responsible for the results we describe here. Thirdly, when discussing methodological flaws, it should also be mentioned that a health based bias of the GNT-HIS participants could be more or less excluded [24], while the design of the OI-survey (anonymously sent back questionnaires) did not allow for evaluation of the non-responders. For that we do not know whether only persons with health problems sought out the opportunity to fill in the questionnaire in order to voice their problems or if persons with health problems in particular did not feel up to this task.

The here conducted explorative analysis found a slightly different risk profile for over-indebted individuals as compared to individuals of the general population. Especially socioeconomic variables, e. g. education or unemployment, seemed to loose influence in the face of strong financial strain like over-indebtedness. In the general population we calculated the same risk profile as reported by other authors [25], namely a decrease in odds ratios with better education and a higher prevalence of back pain in unemployed persons compared to full-time employees. In comparison to that, we could not detect these associations in the participants of the OI-survey. But with a back pain prevalence of 80% in the over-indebted individuals, this result might also be due to a lack of variance in this cohort.

Schneider et al. [26] described in a representative cohort of the German general population (N = 3488 between 18 and 69 years old) occupational categories with lower-than-average and higher-than-average prevalence of back pain, the 7-day-average being 34% in the general German population between 1997 and 1999. What catches the eye in the article of Schneider et al. is the fact that the above-average prevalence was identified for occupations with physically strenuous work and, in most cases, lower socioeconomic class. About 60% of the persons attending debt counselling agencies in Germany are stemming from low socio-economic classes [4] and are therefore likely to hold strenuous jobs. This might have influenced some of the results. Nevertheless, socioeconomic variables like school education and professional education - other possible attributes of lower class manual workers - did not play a statistically significant role in the odds ratios of the OI-survey. This again might be associated with the fact that a large number of over-indebted participants were unemployed.

While regular physical activity for more than two hours a week turned out to be "protective" both in the general population and the over-indebted collective, the calculated OR indicated that the over-indebted persons might profit even more by a regular exercise as their OR with regard to regular activity was calculated to be 0.34 (95%CI: 0.20-.058) compared to 0.70 (95%CI: 0.62-0.79) in the general population. Nevertheless, the little case numbers of over-indebted persons actually working out more than two hours per week have to be considered. Finally, mental illnesses like depression led to a 2.7 times increase in the risk of back pain in the general population but "just" to a 1.8 times increase in the over-indebted population, a result that might depend on a competing effect financial strain has on mental health [12] on the one hand, nevertheless this result might also be due to the different measurement of the variable "mental illnesses" on the other hand.

The available literature indicates a clear link between psychological variables and back pain [27]. Psychological variables are probably related to the onset of pain, and to the occurrence of acute, sub-chronic, and chronic pain [27]. Apart from that, financial strain is probably linked to perceived poor health and depression [12,28-30] and might influence the prevalence of back pain via these mechanisms, too. One factor of financial strain might be measured, amongst other things, by the burden of debt a person faces. This assumption made, over-indebted persons, who are threatened by a life-event like private bankruptcy, seem to be more likely to complain about back pain than the general population. Nevertheless, Skillgate et al. [31] did not find an association between low back pain and life events, but over-indebtedness and bankruptcy were a) not a proposed life event in their examination and b) might not have been important in Sweden at the time of evaluation (1993-1997). In contrast, in our explorative analysis, "over-indebtedness" turned out to be an independent moderator variable factor for the prevalence of back pain. As "over-indebtedness" was not a prompted item in the GNT-HIS questionnaire we coded all GNT-HIS participants (general population) as "not over-indebted" in the combined data set. This procedure might lead to a shift towards the zero-effect and therefore, apart from the methodological discussions above, the calculated ten times higher odds ratio might also depict only a minimum estimation of reality.

## Conclusion

This is the first study that considered over-indebted persons as a special back pain risk group. We found evidence that over-indebted individuals might suffer from back pain more often than individuals from the general population and that over-indebtedness might be an independent moderator variable. The increasing number of over-indebted private households in industrialized countries and the importance of back pain for a countries economy and health care system, gave us reason to believe that a preventive approach to the "public health problem" back pain related to over-indebtedness is imminent. It may be found in socioeconomic, legal and political changes. But a first step in the right direction, i. e. a first step to elucidate the situation, might be the inclusion of a debt anamnesis in longitudinal health surveys of the general population.

## Competing interests

The authors declare that they have no competing interests.

## Authors' contributions

EO and EM had the idea for the evaluation. EO wrote the paper, which was subsequently modified in discussion with all authors. HR, EO, EM were responsible for the analysis. EM and SL conceptualised the OI-survey and developed the study protocol. All authors read and approved the final manuscript.

## Pre-publication history

The pre-publication history for this paper can be accessed here:

http://www.biomedcentral.com/1471-2458/9/451/prepub
